# Perioperative lung function following flow controlled ventilation for robot-assisted prostatectomies in steep trendelenburg position: an observational study

**DOI:** 10.1186/s40635-023-00537-z

**Published:** 2023-08-11

**Authors:** Mustafa Syrafe, Wiebke Köhne, Andre Börgers, Heinrich Löwen, Susanne Krege, Harald Groeben

**Affiliations:** 1 Department of Anaesthesia, Critical Care Medicine and Pain Therapy, Kliniken Essen-Mitte, Henricistr. 92, 45136 Essen, Germany; 2 Department of Urology, Pediatric Urology, and Urologic Oncology, Kliniken Essen-Mitte, Essen, Germany

**Keywords:** Robot assisted surgery, Trendelenburg position, Postoperative lung function, Lung mechanics

## Abstract

**Background:**

Steep Trendelenburg position combined with capnoperitoneum can lead to pulmonary complications and prolonged affection of postoperative lung function. Changes in pulmonary function occur independent of different modes of ventilation and levels of positive end-expiratory pressure (PEEP). The effect of flow-controlled ventilation (FCV) has not been evaluated yet. We perioperatively measured spirometric lung function parameters in patients undergoing robot-assisted prostatectomy under FCV. Our primary hypothesis was that there is no significant difference in the ratio of the maximal mid expiratory and inspiratory flow (MEF50/MIF50) after surgery.

**Methods:**

In 20 patients, spirometric measurements were obtained preoperatively, 40, 120, and 240 min and 1 and 5 days postoperatively. We measured MEF50/MIF50, vital capacity (VC), forced expiratory volume in 1 s (FEV1), and intraoperative ventilation parameters.

**Results:**

MEF50/MIF50 ratio increased from 0.92 (CI 0.73–1.11) to 1.38 (CI 1.01–1.75, *p* < 0.0001) and returned to baseline within 24 h, while VC and FEV1 decreased postoperatively with a second nadir at 24 h and only normalized by the fifth day (*p* < 0.0001). Compared to patients with PCV, postoperative lung function changes similarly.

**Conclusion:**

Flow-controlled ventilation led to changes in lung function similar to those observed with pressure-controlled ventilation. While the ratio of MEF50/MIF50 normalized within 24 h, VC and FEV1 recovered within 5 days after surgery.

## Background

Steep Trendelenburg position in combination with intra-peritoneal gas insufflation, like it is routinely used for robotic assisted prostatectomies, can lead to prolonged postoperative affection of lung function and an increased rate of postoperative pulmonary complications (PPCs) [1–5]. Several studies have evaluated the influence of different modes and settings of ventilation on the rate of PPCs in general [1–3, 6–10]. Neither the comparison of volume-controlled versus pressure-controlled ventilation nor the comparison of high versus low positive end-expiratory pressure (PEEP) during general anaesthesia could detect a difference in PPCs [1, 2, 6–10]. Finally, the investigators of the assessment of ventilation during general anaesthesia for robotic-assisted abdominal surgery (AVATaR) study stated that no ventilatory variables were independently associated with increased occurrence of PPCs, which can be seen in up to 19% [3].

Flow-controlled ventilation (FCV) is conventional available since 2017 and offers some features that differ from standard volume- or pressure-controlled ventilation [11–14]. Besides intratracheally measured PEEP and peak pressure, which allow individually settings guided by compliance measurements, the most prominent difference is a constant, actively controlled, expiratory flow [11–14]. FCV has been evaluated for several experimental settings and its use was associated with improved lung recruitment [12, 14, 15]. FCV has not yet been evaluated for ventilation of patients undergoing the challenging conditions during robot-assisted prostatectomies.

Therefore, to evaluate the effect of FCV on lung function, parameters of intra- and extrathoracic resistance as well as intraoperative ventilation parameters were assessed perioperatively. For the quantification of changes in the extrathoracic airway resistance, as it could be seen following the development of airway oedema due to steep Trendelenburg position, the calculation of the ratio of the maximal mid expiratory and inspiratory flow (MEF50/MIF50) has been established [16]. Increased upper airway resistance compromises inspiration more than expiration, what can clinically be heard as inspiratory stridor. Calculation of this ratio allows to quantify changes over a certain time course.

In addition, we compared the results of this study to results from a historic control. The control patients underwent the same robot-assisted procedure under general anaesthesia with pressure-controlled ventilation (PCV) [5].

Our primary hypothesis was that there is no significant difference in the ratio of the MEF50 divided by the MIF50 after surgery compared to preoperative baseline.

## Methods

### Patients

**Fig. 1:**
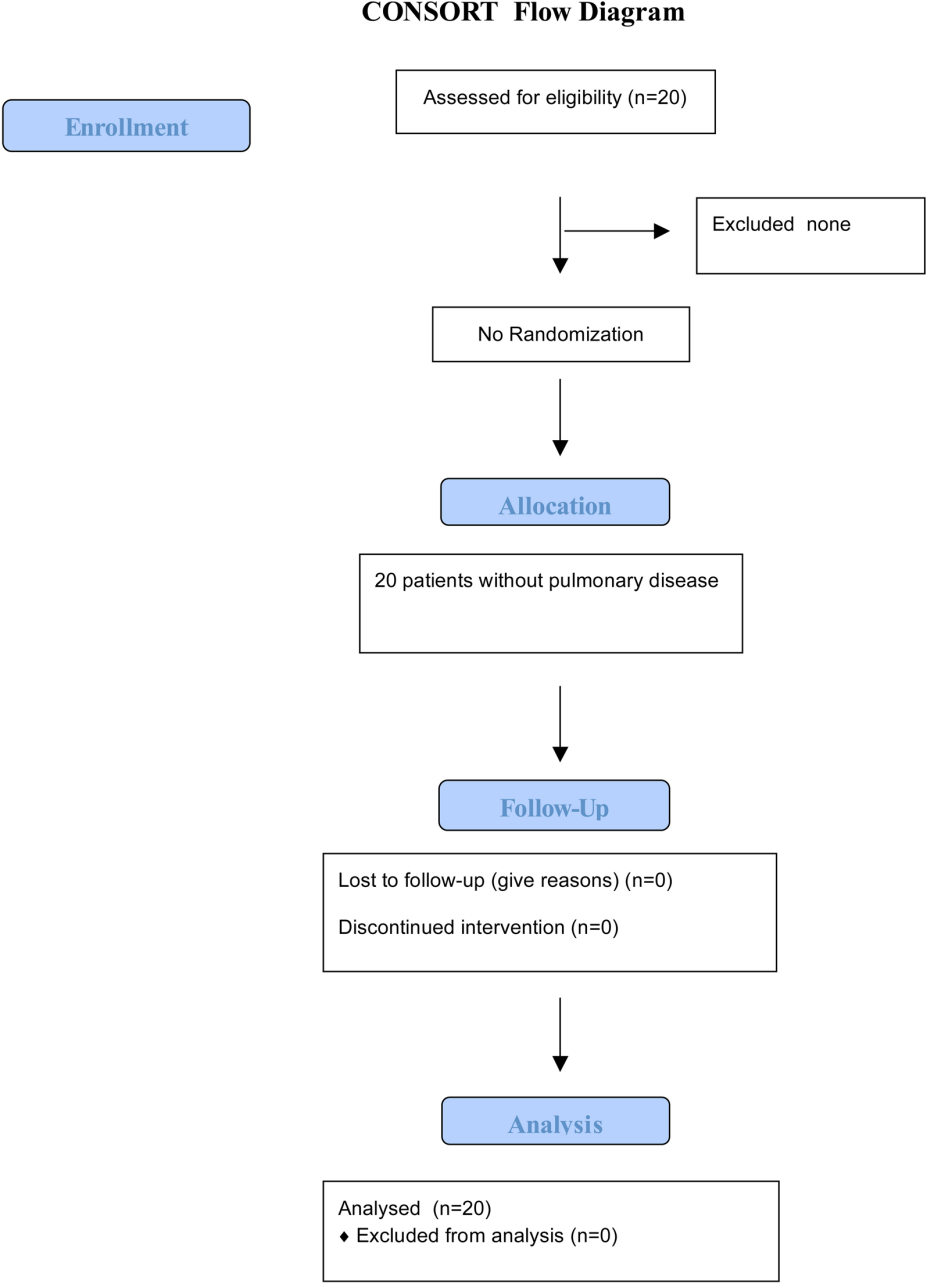
CONSORT flow diagram for the enrolment patients and analysis of study results

The local ethics committee (Chamber of Physicians of Nordrhein, Düsseldorf, Germany, Registration-Number 2019298) approved the study and 20 patients were enrolled (Fig. 1).

All patients were scheduled for robot-assisted radical prostatectomy and lymph node dissection and gave their informed written consent to participate.

None of the patients had a history of significant cardiac diseases or obstructive sleep apnoea syndrome and all patients were free of pulmonary disease. All patients received a combination of general and low thoracic epidural anaesthesia.

Patients of the historic-control underwent the same kind of robot-assisted prostatectomy under PCV and participated in perioperative lung function measurements. All of these patients were free of any cardiac and pulmonary disease and had no history of obstructive sleep apnoea. The results of lung function measurements of these patients were published in 2018 [5].

### Measurements

Lung function measurements were performed with a spirometer (pneumotachograph, VIASIS, Würzburg, Germany). On the day prior to surgery, in all patients baseline vital capacity (VC), forced expiratory volume in 1 s (FEV_1_), maximal expiratory flow at 50 per cent of the vital capacity (MEF50), and maximal inspiratory flow at 50 per cent of the vital capacity (MIF50) were assessed in sitting position.

Subsequent measurements were performed in supine position with the upper body tilted upwards by 40 degrees. According to international guidelines, always the best of three measurements was accepted for analysis [17].

### Protocol

Prior to surgery lung function was measured in supine position before the patients were taken into the induction room. Subsequently, after arrival in the induction room a peripheral intravenous line was started and standard monitoring (ECG, non-invasive blood pressure measurement, fingertip for arterial oxygen saturation) was applied. All patients received a thoracic epidural catheter in sitting position at T9/10 or T10/11 thoracic vertebral interspace in sitting position with loss of resistance technique. After induction of general anaesthesia and during surgery the epidural catheter was dosed with 0.5% bupivacaine solution.

Subsequently, general anaesthesia was induced with propofol, remifentanil, and rocuronium. The dose of the agents were administered according to the discretion of the anaesthetist. The patient’s trachea was then intubated with a Tritube (Ventinova Medical B.V., Eindhoven, The Netherlands) with an outer diameter of 4.4 mm and an internal diameter of 2.3 mm with an integrated channel to measure tracheal pressure at the tip of the tube. Flow controlled ventilation was started with the Evone ventilator (Ventinova Medical B.V., Eindhoven, The Netherlands).

For the titration of an individualised PEEP, ventilation was initiated with a PEEP of 5 cmH_2_O and PEEP was increased under a constant pressure difference of 10cmH_2_O (peak-PEEP) until the tidal volume reached its maximum. Next, peak pressure was stepwise increased until dynamic compliance started to decrease. When this point was reached, the previous peak pressure value was used. Inspiration to expiration ratio was kept at 1:1 and flow was set to achieve end-expiratory CO_2_ pressure between 34 and 40 mmHg. Once, the best individual dynamic compliance was determined and the flow was set, respiratory rate results from this setting.

Since all patients had received an epidural catheter, which was used to administer 0.5% solution bupivacaine according to the height of the patients, no additional systemic muscle relaxing medication was given throughout surgery. However, train of four was controlled at the end of surgery to rule out any residual rocuronium effects.

Robot-assisted prostatectomy was performed with the DaVinci system in 30º Trendelenburg position.

Thirty minutes after extubation, lung function was measured in the recovery room and repeated after 120, 240 min, 24 h, and after 5 days. Prior to lung function measurements, pain score (Numerical Rating Score, with 0 for no pain and 10 for worst imaginable pain) and the presence of chemosis was assessed. Chemosis was described as clinically present or not.

### Data analysis

Data are presented as mean ± 95% confidence intervals or box plots (10., 25., 75., 90. percentile and median). Sample size calculation was based on the primary hypothesis that there is no significant difference in the ratio of the MEF50 divided by the MIF50 with a difference to detect of minimal 0.25, an alpha error of 0.05, a beta error of 0.8 and standard deviation of 0.30. The result was a minimal number of 17 patients. We rounded the number to 20.

In addition two secondary hypotheses were tested. First, there was no perioperative difference in VC within the group of patients. Second, there was no perioperative difference in FEV1 within the group of patients.

In addition, in an explorative approach, results of lung function measurements were compared to a historic control.

The primary hypothesis was tested by ANOVA for repeated measurements followed by a posthoc test with Bonferroni correction for multiple testing. Patients’ characteristics were tested by student t-test. The primary hypotheses was rejected and significant differences assumed with p < 0.05.

The results of the historic control group were compared by Two-Way-ANOVA for repeated measurements.

## Results

### Characteristics of the patients

Characteristics of patients with FCV and the patients of the historic control under PCV including baseline lung function measurements, duration of surgery, and the amount of intraoperative fluid administration are presented in Table 1.

**Table 1:** Patient characteristics, duration of surgery, intravenous fluid administration and predicted vs. baseline Vital Capacity (VC), forced expiratory Volume in 1 s (FEV1) and Oxygen Saturation (SO2) in patients under flow controlled vs. (FCV) pressure controlled ventilation (PCV) (mean; 95% Confidence Interval or Standard Deviation)

	FCV (*n* = 20)	PCV (*n* = 56)	*p*-value
Height (cm; SD)	177 (6)	178 (7)	0.4008
Weight (kg; SD)	85 (13)	86 (12)	0.5760
Age (y; SD)	65 (7)	67 (7)	0.3447
BMI (SD)	27.2 (3.8)	27.4 (3.5)	0.9141
Vcpred. (l; CI)	4.15 (3.86–4.44)	4.31 (4.17–4.45)	0.3045
VCbase. (l; CI)	4.52 (4.15–4.89)	4.55 (4.37–4.73)	0.8615
FEV1pred. (l; CI)	3.13 (2.94–3.32)	3.29 (3.16–3.42)	0.1616
FEV1base. (l; CI)	3.39 (3.08–3.70)	3.38 (3.23–3.54)	0.9601
Surgery (min; CI)	195 (177–213)	210 (198–222)	0.1935
i.v. Fluid (ml; CI)	1840 (1675–2005)	2018 (1899–2136)	0.2471
SO2 (%; CI)	97 (96.8–97.8)	96 (95.7–96.7)	0.1664

There were no statistical differences in the lung function measurements and oxygen saturation.

### Primary outcome measure

**Fig. 2:**
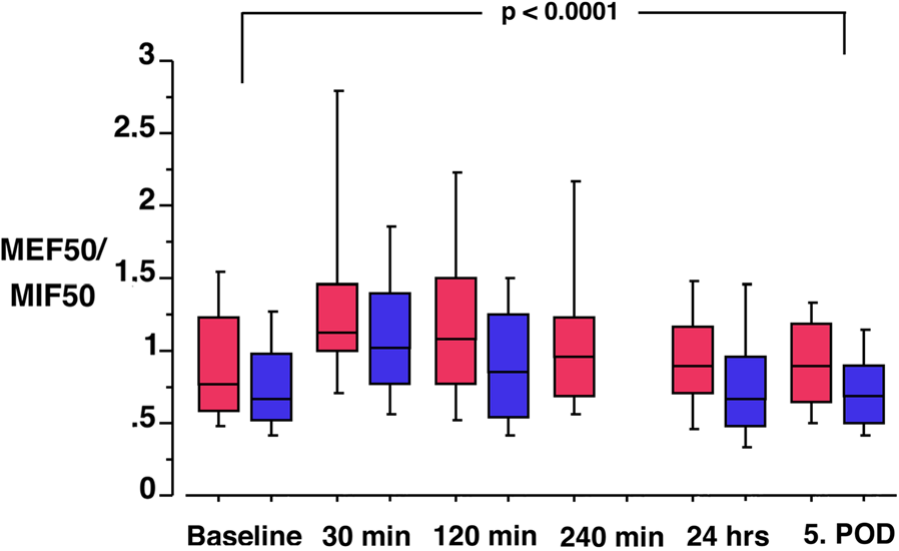
Box plot of the MEF50/MIF50 ratio from baseline prior to surgery to the 5. postoperative day. Red boxes represent patients under flow controlled ventilation (*n* = 20), while blue boxes represent patients of the historic control (*n* = 56). For both groups MEF50/MIF50 ratio increases significantly after surgery and returns to baseline within 24 h. There is no difference between the groups over time (*p* = 0.7745). Significant differences could be found between baseline vs. 30 min (*p* < 0.0001) and 120 min (*p* = 0.0042)

As the primary outcome measurement the MEF50/MIF50 was assessed. In the standard sitting position the ratio was 0.88 (CI 0.72–1.04). There was a significant difference over time with a peak directly after surgery (*p* < 0.0001; Fig. 2). The ratio started prior to surgery with 0.92 (CI 0.73–1.11), increased 30 min after surgery to 1.38 (CI 1.01–1.75; *p* < 0.0001), decreased after 120 min to 1.22 (CI 0.92–1.52; *p* = 0.0042), and after 240 min to 1.10 (CI 0.81–1.39; *p* = 0.682). The next morning the ratio returned to 0.93 (CI 0.75–1.11, *p* = 0.8838) and at the 5. day to 0.92 (CI 0.76–1.08; *p* = 0.9711).

Over time the ratio changed significantly (*p* < 0.0001; Fig. 2). The ratio changed similar in both groups and there was no difference between the groups over the five day time period (*p* = 0.7745; Fig. 2).

### Secondary outcome measures

**Fig. 3:**
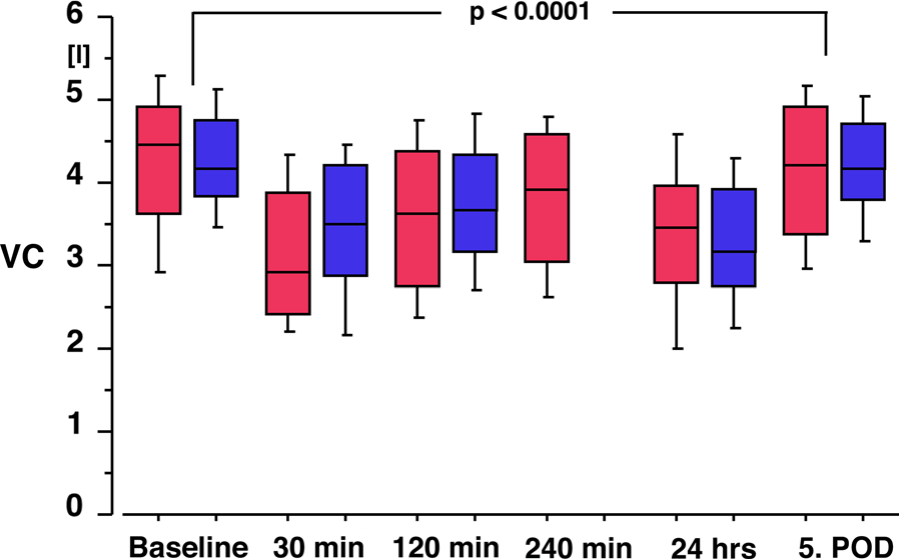
Box plot of the vital capacity (VC) from baseline prior to surgery to the 5. postoperative day. Red boxes represent patients under flow controlled ventilation (*n* = 20), while blue boxes represent patients of the historic group under pressure controlled ventilation (*n* = 56). Vital capacity is significantly decreased after surgery with a second nadir after 24 h and a return to baseline after 5 days. There is no difference between the groups over time (*p* = 0.2222)

**Fig. 4:**
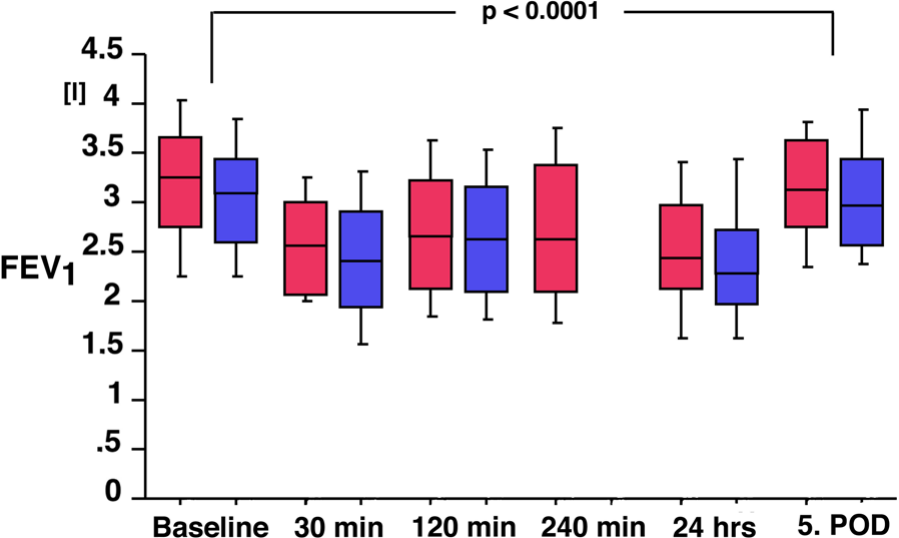
Box plot of the forced expiratory volume in one second (FEV1) from baseline prior to surgery to the 5. postoperative day. Red boxes represent patients, who underwent flow controlled ventilation (*n* = 20), while blue boxes represent patients of the historic group under pressure controlled ventilation (*n* = 56). FEV1 is significantly decreased after surgery with a second nadir after 24 h and a return to baseline after 5 days. There is no difference between the groups over time (*p* = 0.1070)

VC and FEV1 changed significantly with the change in posture from sitting (VC: 4.52 l (CI 4.15–4.89 l); FEV1 3.39 l (3.08–3.70 l) to supine (VC: 4.26 l (CI 3.85–4.67 l); FEV1 3.21 l (CI 2.91–3.51 l; *p* = 0.0001 and *p* < 0.0001, respectively). Compared to preoperative measurements in supine position, VC and FEV1 decreased significantly postoperatively with two nadirs one directly after surgery and the other at 24 h (*p* < 0.0001; Figs. 3 and 4). At the fifth day, mean VC and FEV1 reached 97% (VC) and 98% (FEV1) from preoperative baseline (*p* = 0.1372 and *p* = 0.3171; Figs. 3 and 4), respectively.

Compared to the PCV group VC and FEV1 results were not different over time (*p* = 0.2222 and *p* = 0.1070).

Intraoperatively, patients under FCV were ventilated with a mean PEEP of 7.2 cmH_2_O (CI 6.8–7.6 cmH_2_O) and a mean airway pressure of 23.4 cmH2O (CI 21.8–25.0 cmH_2_O) resulting in a tidal volume of 521 ml (CI 493–549 ml) and a respiratory rate of 12/minute (CI 11.1–12.9 /minute). Mean dynamic compliance was 37.2 ml/cmH_2_O (CI 32.8–40.6 ml/cmH_2_O).

### Oxygen saturation

At the beginning of the measurements prior to forced respiratory manoeuvres the arterial oxygen saturation was measured.

Compared to baseline oxygen saturation decreased significantly postoperatively (*p* < 0.0001) with the lowest values directly after surgery (96.9 ± 1.1% vs. 95.6 ± 1.5%, *p* = 0.0007). Oxygen saturation was still reduced after 24 h (96.9 ± 1.1% vs. 95.8 ± 1.3%, *p* = 0.0012) and recovered to baseline after 5 days.

### Pain score

Pain level of the patients was evaluated prior to each lung function measurement. All patients presented for the lung function measurements with a pain score of 2 or less.

### Pulmonary complications

None of the 20 patients developed clinically relevant complications like pneumonia prolonged oxygen therapy or any specific medication or intervention, because of postoperative pulmonary complications.

### Chemosis

Out of 20 patients, 9 patients presented with mild to moderate chemosis at the end of surgery.

## Discussion

In patients free of pulmonary disease the ratio of MEF50/MIF50 increases after robotic assisted prostatectomies under FCV with a return to baseline within 24 h. In contrast, VC and FEV1 are significantly reduced directly after surgery with a second nadir at 24 h and a recovery to baseline within 5 days. These results are quite similar to the results of a historic group, which has been ventilated for the same procedure with PCV.

Robotic-assisted surgery in steep Trendelenburg position for surgery in the lower abdomen like prostatectomies, hysterectomies, and colo-rectal surgery has gained widespread popularity [17–20]. Compared to open surgery, these types of minimal invasive surgery offer excellent surgical conditions, a fast recovery, and a short hospital stay. However, steep Trendelenburg position in combination with peritoneal gas insufflation creates a cranial shift of the diaphragm and oedema of the extrathoracic airways [2, 4–7]. Therefore, pulmonary compliance and overall pulmonary function can be affected for several days with an increased risk of pulmonary complications, which can reduce the potential benefit of the robotic-assisted procedure [1, 3–5].

Several studies have documented the increased risk of PPCs for patients undergoing procedures in steep Trendelenburg position [1, 3–5]. The effect of pressure-controlled ventilation has been compared to the effect of volume controlled-ventilation, as well as different levels of PEEP [1, 2, 7, 9, 10]. Most recent studies describe better pulmonary compliance under protective ventilation and increased PEEP, but no difference in PPCs, hospital stay, and lung function after 24 h. [1, 3, 7, 10] However, high levels of PEEP (up to 15 cmH_2_O) led to haemodynamic compromise with an increased need for catecholamine support to stabilise perfusion [1, 7]. Furthermore, steep Trendelenburg position has been shown to increase intraocular pressure far above normal upper limits, which potentially increases even further by high intrathoracic pressure due to high PEEP [20, 21]. An increasing number of vision loss in patients, who underwent several hours lasting surgery in Trendelenburg position and capnoperitoneum has been described [22, 23].

Flow-controlled ventilation has become available since 2017 and has not been described for the use in patients under robot-assisted surgery. FCV can be applied via a specially designed tube (Tritube). The Tritube has an innerdiameter of 2.2 mm and an integrated small channel for constant measurement of intratracheal pressure to stir ventilatory flow. In contrast to pressure- and volume-controlled ventilation, FCV employs an active expiration (venturi principle), with an in- to expiration ratio of 1:1 and a constant inspiratory and expiratory flow. PEEP and peak inspiratory pressure are measured intratracheally and ventilator settings are guided by dynamic compliance measurements. Setting of the flow determines minute ventilation [11–13]. FCV has been shown to minimise atelectasis formation in experimental and clinical settings [14, 15]. Weber et al. assessed the effect of FCV vs. PCV in obese patients in a randomised study on regional ventilation distribution and end-expiratory lung volume (EELV). They found increased EELV and improved regional ventilation distribution under FCV compared to VCV [15].

Since, the effect of FCV in robot-assisted surgery has not been described yet, our intention was to evaluate the effect of FCV on perioperative lung function and intraoperative ventilation. Besides the observational aspect of the study, we compared the results to a historic group of patients, who also underwent perioperative spirometry and robot-assisted prostatectomy, ventilated under PCV.

In our study, individual PEEP and peak inspiratory pressure were determined and applied to ventilate at the best dynamic compliance based on tracheal pressure measurements. In fact, the mean PEEP applied was only slightly higher than the PEEP level that was defined as low PEEP in several recent studies [1, 3, 8, 10]. Tidal volume remained within the range of 6 to 8 ml per kg ideal body weight. However, there were no clinically relevant pulmonary complications that required specific intervention or prolonged hospital stay of the patients. The lack of PPCs can be explained by the rather practical definition and the fact that all patients were free of pulmonary disease to begin with. Moreover, some studies apply different definitions of PPCs and define already an unplanned use of oxygen application after surgery as a complication [3, 8].

Concerning spirometric lung function measurements, we found an increase of the upper (extrathoracic) airway resistance expressed as a significant increase of the MEF50 to MIF50 ratio. The affection of the upper airway can be explained by mucosal oedema, due to venous congestion by extreme positioning, initiation of a capnoperitoneum and increased intrathoracic pressure according to mechanical ventilation. Since, peak and mean airway pressure may differ depending on the mode of ventilation, FCV may lead to lower intrathoracic pressure compared to PCV and thus influence oedema formation and changes in MEF50 to MIF50 ratio.

Accordingly, this affection resolves within hours with reversed positioning of the upper part of the body and return of spontaneous breathing and leads subsequently to the resolution of oedema. We described the same effect in our previous studies under PCV [4, 5]. Therefore, our results support again the recommendations for a restrictive intraoperative fluid management.

Moreover, the patients developed a significant reduction in VC and FEV1, which exceeded the changes in upper airway resistance by several days. After an initial reduction in VC and FEV1 both spirometric parameters improve markedly within the first hours, but go through a second nadir in the first postoperative night [3, 4]. This phenomenon has been well described before [24]. We can only speculate about the reason behind that. Most likely, some hypoventilation during sleep combined with residual atelectasis after mechanical ventilation led to a second nadir of VC the morning after surgery. Residual atelectasis for more than 24 h have been described before. [25]

Finally, VC and FEV1 returned to 97% and 98% of preoperative baseline until the fifth postoperative day. This effect seems to develop independently of the ventilation technique. Shono et al. describe a reduction in VC and FEV1 of 27 to 33% 24 h postoperatively, regardless whether PCV with protective ventilation or VCV was applied [1].

In our previous studies, we found the same effect under PCV [4, 5]. Comparing our findings under FCV to the results of the historic control, we could not find a difference between patients ventilated with FCV or PCV. The results of lung function measurements follow the same pattern independent of the technique of mechanical ventilation.

There are several limitations to our study. It can be argued that postoperative lung function measurements can be influenced by reduced vigilance, residual muscle relaxation and pain of the patients.

First, spirometric lung function measurements rely on the cooperation of the patients and can be altered by postoperative effects of general anaesthesia. However, based on previous studies, we felt confident that patients, who were able to follow simple commands after removal of the endotracheal tube, will be able to perform deep breath and forced manoeuvres 30 to 40 min later. In particular, all patients who received a combined general and epidural anaesthesia were expected to be awake enough [4, 5, 26].

Second, residual neuromuscular blockade could be excluded because all patients received rocuronium only at the induction of anaesthesia, which was about 240 min prior to the spirometric measurements. Nevertheless, to exclude residual neuromuscular blockade acceleromanometry was performed at the end of surgery and showed percentages of 97% and more [26].

Third, postoperative pain might have led to incomplete spirometric manoeuvres of the patients. Therefore, a pain score was assessed prior to each lung function measurements. Measurements were performed only when patients described their pain with a pain score of less than 3 following a deep breath [27].

Another limitation of our study is related to the comparison of the actual and the historic group. The two groups of patients were not randomised and this explorative approach was not the primary outcome this study was powered for. However, with the standardisation of the surgical and the anaesthetic management of the procedure and the only difference in the ventilation technique, we felt comfortable to compare these groups. All patients were free of pulmonary disease and presented with normal oxygenation. In particular patients of the two groups did not differ in most of the typical risk factors for postoperative pulmonary complications as described by Fernandez-Bustamente et al.: Emergency surgery, surgical site, age, preoperative oxygenation, anaesthesia duration, and body mass index [28]. Overall, we felt comfortable to compare these two groups [28].

In conclusion, our results show postoperative lung function and incidence of PPCs seem not to be influenced by the mode of ventilation in a lung healthy study population. Although FCV offers unique ventilation features, that had led to significantly improved gas exchange and lung tissue aeration intraoperatively in previous studies, we could not detect a significant difference compared in postoperative lung function compared to the historic control under PCV. MEF50/MIF50 normalized within 24 h, while VC and FEV1 were still significantly decreased after 24 h and reached almost 100% of their preoperative baseline values at the fifth postoperative day.

## Data Availability

The datasets used and analysed for the study are available from the corresponding author on reasonable request.

## Abbreviations

ANOVA: Analysis of variance
CI: Confidence Interval
DC: Dynamic compliance
ECG: Electrocardiogram
EELV: End-expiratory lung volume
FCV: Flow-controlled ventilation
FEV1: Forced expiratory volume in 1 s
MAP: Mean airway pressure
MEF50: Maximal mid expiratory flow at 50% of VC
MIF50: Maximal mid inspiratory flow at 50% of VC
MV: Minute ventilation
PEEP: Positive end-expiratory pressure
PCV: Pressure-controlled ventilation
PPC: Postoperative pulmonary complications
TV: Tidal volume
VC: Vital capacity
VCV: Volume-controlled ventilation
